# Effectiveness of pharmacological procedural sedation in children with cerebral palsy undergoing botulinum toxin injection: a systematic review and meta-analysis

**DOI:** 10.3389/fped.2025.1610064

**Published:** 2025-09-03

**Authors:** Veronica Lazzaretto, Egidio Barbi, Carolina Carraro, Marco Sutera, Fulvio Celsi, Francesca Peri, Luisa Zupin

**Affiliations:** ^1^ University of Trieste, Trieste, Italy; ^2^ Department of Pediatrics, Institute for Maternal and Child Health, IRCCS Burlo Garofolo, Trieste, Italy

**Keywords:** cerebral palsy, botulinum toxin injection, procedural sedation, spasticity, sedation

## Abstract

Botulinum toxin injection (BTI) is a well-established therapeutic option for focal spasticity in children with cerebral palsy (CP). However, the procedure is associated with significant anxiety, pain, and discomfort, highlighting the importance of using sedation and distraction techniques. To date, no specific guideline exists for procedural sedation during BTI in children with CP. Therefore, we conducted a systematic review and meta-analysis to investigate the strategies for improving the experience of children with CP during BTI. Articles published up to July 2024 were identified through PubMed searches, and after screening, ten studies involving children aged 0–18 with CP were selected. Pain levels during BTI procedure was assessed using specific pain scales. Nitrous oxide was used as a sedation treatment in 7 studies, either alone (3 studies), combined with topical anaesthetic (3 studies), or alongside both topical anaesthetics and distraction techniques (2 studies). Its use reduced distress, with the greatest effect when combined with topical anaesthetic. These results suggest that the combination of pharmacological and non-pharmacological methods may be a reasonable approach. The most common limitations of the studies included were the absence of a clear definition of the degree of cognitive impairment, the lack of dedicated pain assessment scales, insufficient consideration of caregiver involvement, and of non-pharmacological techniques. Further research is needed to establish standardized guidelines for procedural sedation during BTI in children with CP, focusing on randomized controlled study designs, precise population definitions, the effectiveness of distraction techniques, caregiver involvement, and the use of dedicated pain assessment scales.

## Introduction

Cerebral palsy (CP) is a permanent, non-progressive disorder of motor function and posture caused by abnormalities of the developing fetal or infant brain. A large proportion of these children present some form of intellectual disability, impairment of speech and vision, or behavioural difficulties, while epilepsy occurs in 20%–40% of the patients (1). There is strong evidence that children with CP and developmental disability experience pain more often than their healthy peers. Those with the most severe cognitive impairment suffer the highest level of pain, which further diminishes their quality of life (2). In addition to common causes of pain, these children experience acute pain due to constipation, multiple caries, esophagitis deriving from untreated gastroesophageal reflux. They are also susceptible to iatrogenic pain resulting from surgical interventions, as well as diagnostic and therapeutic procedures. Moreover, children with CP frequently suffer from chronic pain, primarily related to muscle spasms, skeletal deformities, and joint dislocations caused by spasticity syndrome, experienced by approximately 80% of them. To date, several approaches are available to treat spasticity, including physical therapy, pharmacological treatments, and surgical interventions (3). The choice of treatment depends on the specific pattern of spasticity presented by each patient (generalized, segmental or focal) and different interventions are often combined. Intramuscular botulinum toxin injection (BTI) is an established treatment in the pharmacological management of focal spasticity (4). Once injected in the selected muscle, botulinum toxin type A prevents the release of acetylcholine from the pre-synaptic nerve terminal, thus blocking peripheral cholinergic transmission at the neuromuscular junction (NMJ). This results in a reduction of muscle contraction, lasting for 3–4 months. The procedure could be performed either on a single muscle or on multiple sites, and a variety of localization techniques is available to assist correct placement of the injection, including electromyography (EMG), nerve or muscle stimulation and imaging, using ultrasound or occasionally, computed tomography (CT) scanning.

Botulinum toxin injection has been widely used for over 20 years during which time it has proved to be a safe treatment option (5). Complications are uncommon and include muscle weakness, transient flu-like symptoms, and more rarely subclinical autonomic dysfunction, dysphagia and respiratory failure when injected in the cervical area. The effectiveness of BTI on focal spasticity in children with CP has been demonstrated by several studies, improving gait, posture and mobility, minimizing contractures and deformities, and reducing pain from spastic syndrome (6). However, intramuscular injection is painful itself and is associated with high levels of anxiety. Moreover, most patients must undergo repeated and multiple injections to maintain an acceptable level of efficacy. Therefore, it is essential to set adequate procedural sedation in order to contrast pain and fear in children with CP undergoing BTI. Many valid approaches exist, from general anaesthesia to conscious sedation or non-pharmacological distraction techniques (7, 8). However, international medical societies have not published guidelines for managing procedural pain and anxiety related to BTI in children with CP. This lack of standardization may be partly attributed to the off-label use of various sedative and analgesic agents in paediatric patients (9).

Furthermore, the implementation of procedural sedation by paediatricians in the clinical practise may face several challenges, particularly in resources-limited settings or rural areas (10). These procedures should be performed exclusively in hospital settings, as they required dedicated and trained healthcare personnel, appropriate monitoring equipment, and the availability of emergency rescue options (11).

Only one narrative literature review on this topic was published in 2021 (12), and it was limited by the need for a systematic approach, the lack of detailing the tools used to measure pain in children, and the possible addition of non-pharmacological techniques. The authors concluded that there was insufficient evidence to identify a superior strategy for reducing pain and anxiety related to BTI in children with CP. Furthermore, no meta-analysis has been conducted to date to summarize the existing data.

In this work, we performed a systematic review and a meta-analysis to evaluate the effectiveness of current pharmacological regimens of procedural sedation in children with CP undergoing botulinum toxin injection.

## Materials and methods

### Literature search and article selection

This systematic review and meta-analysis was conducted following the PRISMA (Preferred Reporting Items for Systematic Reviews and Meta-Analyses) statement. Two investigators searched PubMed (https://pubmed.ncbi.nlm.nih.gov) up to July 2024, employing a combination of terms related to sedation during botulinum toxin injections in paediatric patients with cerebral palsy. We included records in English, Spanish, and French. The investigation utilized the following key phrases: (“sedation” AND “botulinum toxin” AND “cerebral palsy”), (“pain management” AND “botulinum toxin” AND “cerebral palsy”), (“midazolam” AND “botulinum toxin” AND “cerebral palsy”), (“ketamine” AND “botulinum toxin” AND “cerebral palsy”), (“nitrous oxide” AND “botulinum toxin” AND “cerebral palsy”), (“propofol” AND “botulinum toxin” AND “cerebral palsy”), (“local anaesthetics” AND “botulinum toxin” AND “cerebral palsy”), and (“non-pharmacological therapies” AND “botulinum toxin” AND “cerebral palsy”). Initially, the titles and abstracts of potentially eligible studies were manually screened for relevance, followed by full-text retrieval for detailed evaluation. Two investigators independently assessed each article for eligibility and resolved any disagreements through consensus.

Inclusion criteria:

- Clinical studies on human subjects with cerebral palsy
- Paediatric age of subjects (0–18 years)
- Use of sedation to perform botulinum toxin injection (BTI)
- Use of methods of distraction to perform BTI
- English, Spanish and French language
- Peer-reviewed paper

Exclusion criteria:

- Adult patients over 18 years with cerebral palsy
- Patients undergoing general anaesthesia
- *In vivo* and *in vitro* pre-clinical studies
- Other languages

### Meta-analysis

We employed Rstudio free software and the “meta” package to conduct the meta-analysis. The outcome employed in the meta-analysis was the level of pain (mean ± standard deviation), mainly assessed through the FLACC and VAS pain scale, during the treatment. Studies were categorized based on the type of sedation procedure employed. The “meta mean” function was used to calculate the overall mean pain level across study groups applying the inverse variance method for pooling. The results were presented using the random effect model (REM) with 95% confidence interval (CI). Statistical heterogeneity was assessed by *I*^2^ and *τ*^2^ statistics, and was reported in the forest plot, which also included the overall REM estimate across all the studies. The influence of individual studies on the meta-analysis was evaluated using the “meta-inf” function.

The assessment of the risk of bias was conducted by 4 authors independently, following the NHLBI (National Heart, Lung and Blood Institute) Study Quality Assessment Tool for Quality Assessment of Controlled Intervention Studies and Quality Assessment Tool for Observational Cohort and Cross-Sectional Studies available at https://www.nhlbi.nih.gov/health-topics/study-quality-assessment-tools. Each item was evaluated as numerically as low (0), moderate (1), high (2) or critical (3), and an overall judgment was measured, resulting in 4 levels: low = 0–10 (0%–25%), moderate = 11–21 (25%–50%), high = 21–31 (50%–75%) and critical = 32–42 (75%–100%). Substantial discrepancies between the authors were resolved through consensus, while the average classifications of all authors were summarized.

We graphically presented the results using the “robvis” online tool (https://mcguinlu.shinyapps.io/robvis/) and evaluated the certainty, following the Grading of Recommendations, Assessment, Development, and Evaluations (GRADE) framework (available at guidelinedevelopment.org/handbook).

## Results

### Meta-analysis

**Figure 1:**
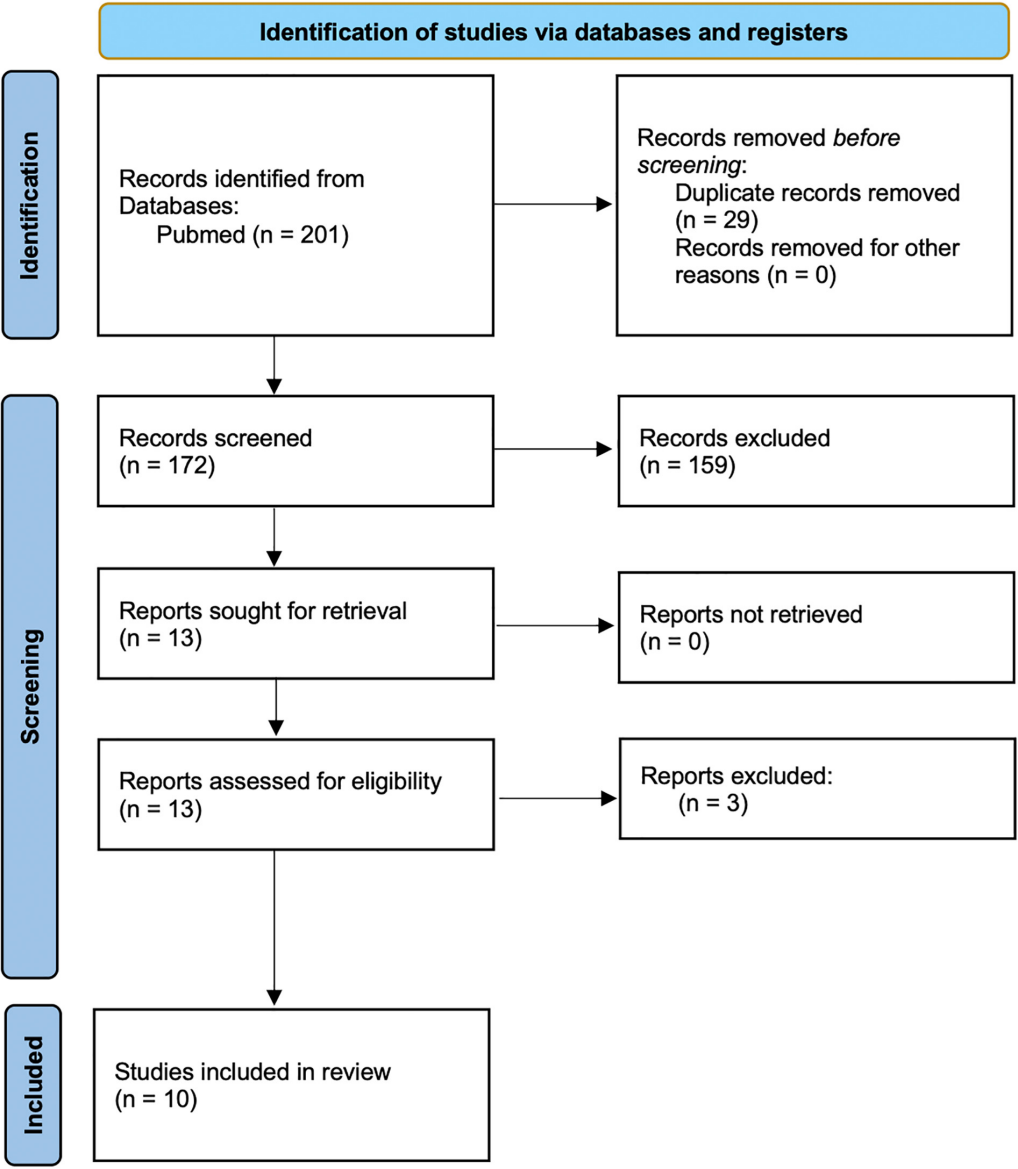
Flow diagram of the systematic review.

The search identified 201 records. Following the removal of duplicates, and after title and abstract screening, and full-text reviews, ten studies were selected for the meta-analysis (Supplementary Table 1) (13–21) and 4 excluded (Figure 1). We ruled out Forrester (30) and Gambart (31) since they did not use a standard pain evaluation scale and omitted Rivard et al. (32) because they only reported pain pre- and post-procedure without data during the procedure. Ostoijc (33), was excluded as it was the only one with biofeedback assisted relaxation training (BART) and distraction therapy.

Nitrous oxide was applied for sedation treatment in 7 studies, either alone (in 3 studies), in combination with topical anaesthetic (in 3 studies), or alongside both topical anaesthetic and distraction techniques (in 2 studies).

The pharmacological approach was commonly used for sedation treatment. Three studies adopted benzodiazepine (midazolam in 2 studies), while ketamine was chosen in a single study. Additionally, oral sedative was administered used without specifying the specific agent.

Non-pharmacological methods such as cooling and topical anaesthetics were also utilized.

Various pain evaluation scales were used to assess the studies, including FLACC (Face, Legs, Activity, Cry, Consolability, in 6 studies), VAS (Visual Analogue Scale, in 4 studies), and CHEOPS (Children's Hospital of Eastern Ontario Pain Scale, in 2 study), when possible the scale were standardized and converted to a 0–10 point scale for comparability of pain measurement across the different studies.

Since most of the studies are observational cohort studies, the analysis focused on investigating pain levels during sedation, categorized according to the sedation procedure employed.

The use of nitrous oxide was correlated with the lowest level of distress (raw mean difference—MRAW 3.69; 95% CI 2.53–4.84), especially when combined with topical anaesthetic (MRAW 2.60; 95% CI 1.84–3.36). The combination of nitrous oxide with topical anaesthetic showed medium heterogeneity (*I*^2^ = 61%, *τ*^2^ = 0.27) and in the group employing nitrous oxide alone (*I*^2^ = 53%, *τ*^2^ = 0.52). When the distraction was also added to nitrous oxide and topical anaesthetic, the level of pain was similar to the use of nitrous oxide alone (MRAW 3.30; 95% CI 0.09–6.50), although the heterogeneity was higher in this group (*I*^2^ = 89%, *τ*^2^ = 4.78).

The utilization of cooling was associated with higher levels of pain across the studies (MRAW 5.76, 95% CI 2.04–9.48) respect to the use of nitrous oxide, and in this case, the heterogeneity was high (*I*^2^ = 98%, *τ*^2^ = 7.06). Intriguingly, the use of benzodiazepines presented high level of pain (MRAW 5.16, 95% CI 4.12–6.20) and a low-medium heterogeneity (*I*^2^ = 33%, *τ*^2^ = 0.27).

Finally, the use of topical anaesthetic demonstrated an intermediate level of ache (MRAW 4.16, 95% CI 3.31–5.02) with low heterogeneity (*I*^2^ = 0%, *τ*^2^ = 0).

**Figure 2:**
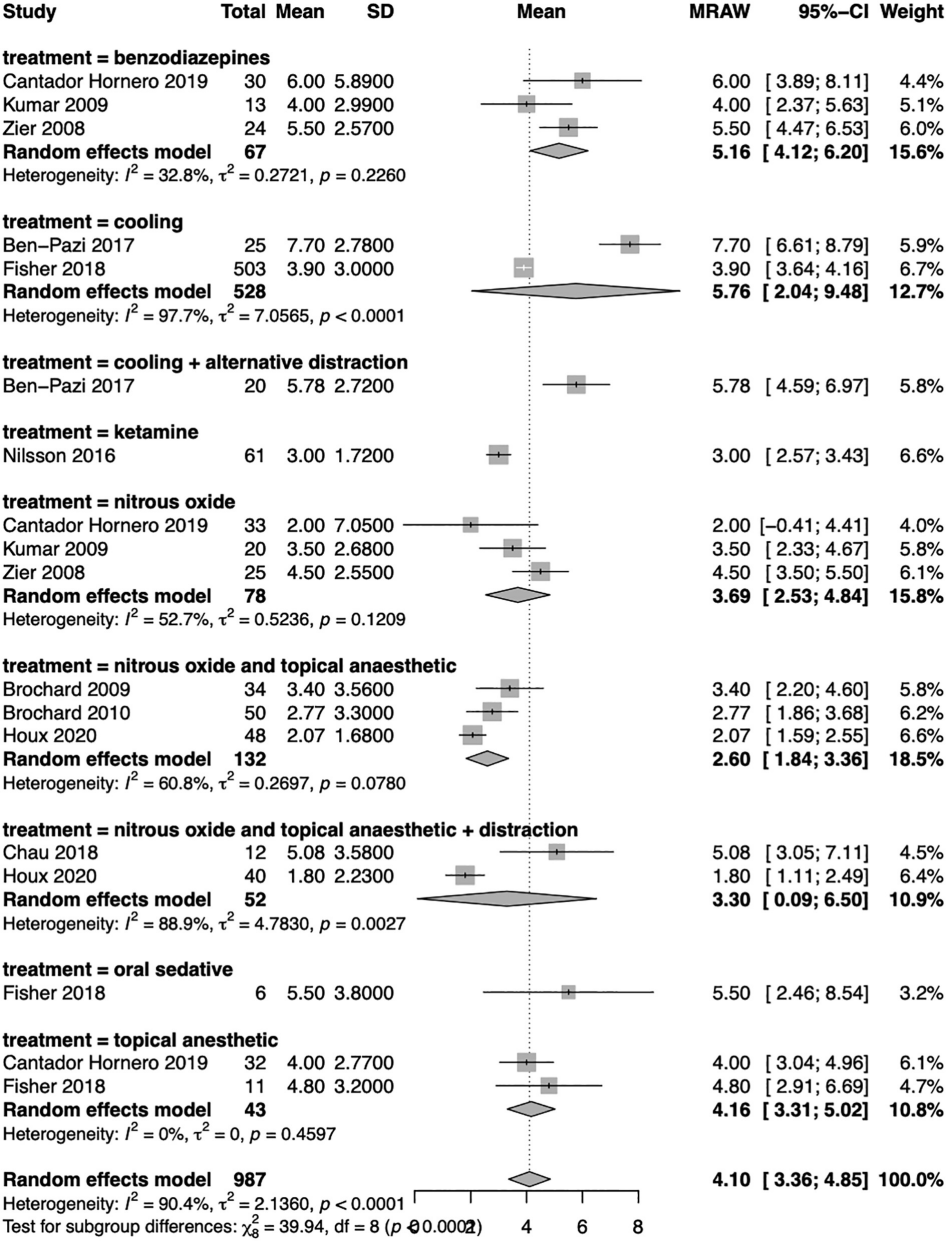
Forest plot of the meta-analysis. The subgroup analysis was conducted based on the technique employed to perform sedation. Random effect model results with raw mean difference (MRAW) and 95% confidence interval (CI) were reported along with heterogenicity assessment (*I*^2^ and *τ*^2^).

Only one study involved cooling plus alternative distraction; another used ketamine, and a third used an oral sedative, so they were inserted in the analysis, but a comparison was not possible (Figure 2).

The influence analysis did not reveal any study that greatly influenced the total result (Supplementary Figure 1).

### Risk of bias and grade of evidence

**Figure 3:**
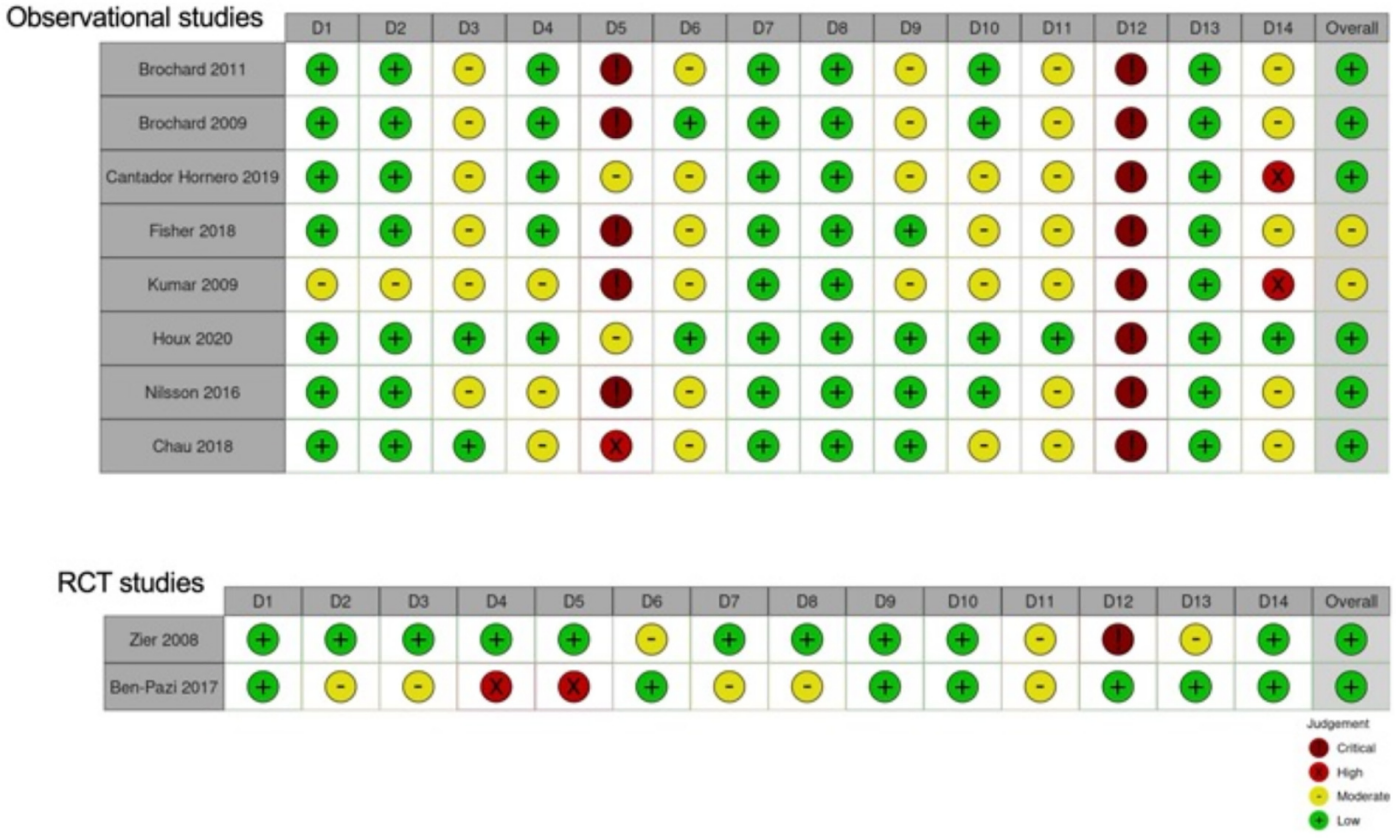
Risk of bias visualization divided in observational studies and RCT studies.

The risk of bias was evaluated as “low” in the 2 RCT studies, as in most of the studies conducted on observational cohorts. However, in this last group, two studies were classified with a moderate risk of bias. The most critical issues in the observational studies were items 5 and 12. Specifically, most examinations did not report justification of the sample size, and the outcome assessors were not blinded to the procedure (Figure 3).

The lowest pain levels were observed in treatments combining nitrous oxide with topical anesthetics. The certainty of evidence for this approach was rated as low-moderate, primarily due to heterogeneity across the studies, the presence of moderate risk of bias in two studies and the predominance of observational designs (Supplementary Table 2).

## Discussion

This systematic review on procedural sedation for BTI in children with cerebral palsy suggests that commonly employed pharmacological methods for sedation and analgesia are reasonably effective in managing injection-related pain and anxiety. However, the limited number of randomized controlled trials and the observational nature of most of the studies selected do not allow any firm conclusion on which approach should be chosen.

Nitrous oxide was the preferred agent in 7 studies included in the meta-analysis, and it emerged to be a safe and effective agent both as a standalone therapy (3 studies) or in combination with topical anaesthetic (3 studies) and/or distraction techniques (2 studies). Treatment with nitrous oxide was associated with low levels of pain. Moreover, the combination of nitrous oxide with topical anaesthetics indicated the highest effectiveness, providing the lowest overall pain level, thus clearly suggesting that an add-on, in terms of analgesia, should always be added to topical anaesthesia.

Benzodiazepines were the second most frequently utilized pharmacological agents, reported as a monotherapy in 3 studies analysed, followed by intravenous ketamine as a single agent, in one study. Surprisingly, the use of benzodiazepines as the sole medication was associated with high levels of distress.

Topical anaesthetics are slightly more effective than benzodiazepines. However, patients receiving this treatment experienced higher levels of pain than individuals treated with nitrous oxide.

Cooling alone was reported in 2 studies and correlated with the highest pain level across the studies.

Single studies considered other techniques, making comparisons challenging. Only one research utilized ketamine, which correlated with a low level of pain, and another work employed an oral sedative (without specifying the drug), also associated with a high level of pain. Only one study evaluated the distraction techniques combined with cooling, and the distress level reported was high, while their use alone was not assessed. Considering these results, a limitation of this method may be related to the difficulty of adequately tailoring distraction techniques to patients with cognitive impairment and limited distraction capacities. While proof from the literature reveals that this population experiences fear and anxiety, which may worsen the painful experience (22), no data are available suggesting the best possible non-pharmacological approaches (23). However, both available evidence and clinical practice suggest that these patients deserve extra care in terms of comfort and distraction technique due to their limited capacities of understanding the explanations, rationale and benefits of the procedure they are going to receive.

Some possible biases in the various studies should also be highlighted. A significant percentage of the analysed studies needed a clear definition of the population involved in verbal performance and gross motor function levels (GMF), making comparisons difficult.

Five out of 10 studies reported the GMF level. Because of this it is also impossible to define whether appropriate pain scales were used in all the analyses. When dealing with nonverbal children with CI a specific pain scale, such as the FLACC revised or the NCCPC-RV, is mandatory to properly assess and measure pain during procedures (24, 25). As a matter of fact, none of the papers evaluated used dedicated scales.

Another methodological issue was the lack of evaluation regarding the impact on patients with the venous access placement, a procedure associated *per se* with more significant pain and anxiety in children with CI than healthy peers.

Another issue the available papers miss, concerns the role of the caregiver, which is not considered in the setting of distraction techniques synchronous to the use of nitrous oxide or topical anaesthesia. Finally, an important but often missing piece of information concerns the number of botulinum injections per patient, as this information is essential to evaluate the duration of the procedure and determine the most appropriate sedation to choose.

In our analysis studies involving general anaesthesia were excluded. While general anaesthesia may offer certain advantages, such as enabling more accurate injection in the desired site, it may be associated with potential risk, including respiratory depression, a side effect that may be serious in children with CP (26, 27). Therefore, the use of general anaesthesia in this population should be carefully evaluated and closely monitored. Moreover, the access to general sedation requires dedicated staff, additional resources, more time and higher cost compared to procedural sedation (28).

### Limitations

The current literature on analgesia and sedation modalities for botulinum toxin injections in children with neurocognitive impairment is limited, with only one previous narrative review available (16). A crucial limitation of our meta-analysis is the small number of published studies (10 in total), which may report only a small fraction of the procedures performed daily in children with CP. However, our analysis included data from over 1,000 patients, with one study enrolling 503 patients and the other ranging from 12 to 54 participants. Another limit is the predominance of observational research, which may impact the conclusions’ strength, indeed eight studies were conducted on CP patient's cohort. Only two works were randomized controlled trials (13, 21) that may represent the gold standard approach to pharmacological clinical studies. However, considering only these studies, they were too few to conduct a meta-analysis. The limited number of works also affected the type of meta-analysis conducted. As a result, we could only provide a descriptive overview of pain levels across the various sedation regimens used without performing a statistical comparison between the different groups.

Additionally, we included investigations with varying pain evaluation scales. Most of them were not ideally suited for non-verbal children, potentially leading to an underestimation of distress levels. Our analysis ruled out cases without standardized pain scales or pain reports during the procedure.

This variability was further evidenced by the moderate to high heterogeneity observed in the meta-analytic statistics.

## Conclusion

By combining both observational and RCT studies we aimed to provide a meaningful, albeit incomplete, overview of the regimens currently employed in procedural sedation for BTI in children with cerebral palsy. Multiple drug regimens are currently in use for procedural sedation for BTI in children with CP. Evidence from the literature suggests that the various sedation techniques applied are reasonably effective across various approaches. Considering the traumatic experience that patients with spasticity encounter due to repeated and multiple botulinum toxin injections and the possible difficulties and impact of venous cannulation in these patients, effective non-intravenous (IV) regimens seem to be a preferable alternative. Moreover, procedural sedation outside the operating room may offer additional advantages, requiring minimal monitoring and fewer resources, with no need for extended post-procedural observation (29). Available data from this review suggest that a reasonable approach could be using nitrous oxide associated with topical anaesthetics, possibly associated with distraction techniques. The limited available evidence suggests that this approach may be more effective than others, more straightforward to deliver, and more acceptable from parents’ and patients’ perspectives. Complex approaches, such as intravenous ketamine, should be reserved for patients in whom the first-line attempt has failed.

### Future trends

Further studies on this topic are needed to establish standardized guidelines for procedural sedation during BTI in children with CP. In order to improve the quality of the research, further investigations should be focused on adopting RCT approach, and more clearly define the study population, paying close attention to factors such as gross motor function and the proportion of verbal vs. non-verbal children. Moreover, distraction techniques should be evaluated, being a cornerstone in analgo-sedation procedures and the caregivers' role should be considered and evaluated in alleviating anxiety in these patients. Finally, the appropriate use of dedicated pain assessment tools for children with neurocognitive impairment, such as the revised FLACC scale, is necessary to ensure an accurate pain evaluation.
