# Supplementary material for: Effectiveness of pharmacological procedural sedation in children with cerebral palsy undergoing botulinum toxin injection: a systematic review and meta-analysis
Source: Front Pediatr. 2025 Sep 3;13:1610064. doi: 10.3389/fped.2025.1610064 (PMC12440712; doi:10.3389/fped.2025.1610064)
Supplement: Supplementary file 1 [file Table1.pdf]

ARTICLE	TYPE OF STUDY	POPULATION (age mean±standard deviation)	SEDATION TREATMENT	ROUTE OF ADMINISTRATION	PAIN SCALE (range)	OUTCOME: LEVEL OF PAIN
Ben-Pazi 2017	RCT	n=45 (7.04±4.68 years)	Cooling	Topical	VAS (0-5)	Cooling+distraction significally reduces pain compared to cooling alone
			Cooling + distraction (clown)	Topical		
Brochard 2009	Observational	n=34 (5.94±4.21)	Nitrous oxide, lidocaine/prilocaine	Inhalation/topical	VAS (0-10)	Nitrous oxide and topical lidocaine/prilocaine may be a reasonable approach
					CHEOPS (4-13)	
Brochard 2010	Observational	n=50 (6.6±4.32)	Nitrous oxide, lidocaine/prilocaine	Inhalation/topical	CHEOPS (4-13)	Children with more severe cognitive impairment seemed to perceive higher levels of pain than the others. Other clinical factors were not associated with pain scores
Cantador Hornero 2019	Observational	n=124	lidocaine/prilocaine	Topical	FLACC (0-10)	While general anesthesia resulted in the lowest levels of pain, using topical anesthesia or nitrous oxide appears to be a reasonable alternative.
			Nitrous oxide	Inhalation		
			Deep sedation	Intravenous		
			Benzodiazepines	Oral/rectal		
Chau 2018	Observational	n=14 (7.79±2.39 years)	Distraction (VR)		FLACC (0-10)	VR was helpful in reducing procedure-related discomfort in a majority of patients.
Fisher 2018	Observational	n=284 (9.2±5.6)	Vapo-coolant	Topical	Not reported, probably VAS (0-10)	Patients treated with topical anesthesia experienced the lowest level of pain during procedure
			No vapo-coolant			
			Lidocaine/prilocaine	Topical		
			Oral anesthetics	Oral		
Houx 2020	Observational	n=59	Nitrous oxide, lidocaine/prilocaine, distraction (music, videos,..)	Topical/inhalation	VAS (0-10)	Distraction with professional clowns did not reduce pain or anxiety more than usual distraction techniques
			Nitrous oxide, lidocaine/prilocaine, distraction (clown)		FLACC (0-10)	
Kumar 2009	Observational	n=33 (8,1±4,9)	Midazolam	Oral	FLACC (0-10)	Pain score in the nitrous oxide group were slightly lower than in the midazolam group
			Nitrous oxide	Inhalator		
Nilsson 2016	Observational	n=61 (5.7±?)	Midazolam, Ketamine	Rectal	FLACC (0-10)	Rectally administered midazolam and racemic ketamine may be a useful alterantive to inhalation analgesia
Zier 2008	RCT	n=50 (8.2±4.4)	Nitrous oxide	Inhalation	FLACC (0-10)	Nitrous oxide produced a level of sedation comparable to midazolam, however patients
			Midazolam	Oral		
