# Supplementary material for: Effectiveness of pharmacological procedural sedation in children with cerebral palsy undergoing botulinum toxin injection: a systematic review and meta-analysis
Source: Front Pediatr. 2025 Sep 3;13:1610064. doi: 10.3389/fped.2025.1610064 (PMC12440712; doi:10.3389/fped.2025.1610064)
Supplement: Supplementary file 2 [file Table2.pdf]

**Certainty assessment**							**Impact**	**Certainty**
№ of studies	**Study design**	**Risk of bias**	**Inconsistency**	**Indirectness**	**Imprecision**	**Other considerations**		
Outcome: pain level								
**10**	8 observational studies and 2 RCT	Low in 7 studies and moderate in 2 studies	moderate due to heterogeneity	not serious	not serious	none	The combination of nitrous oxide and topical anesthetic was associated with the lowest level of pain	Low-Moderate due to inconsistency and risk of bias

Table supplementary 2: Certainty assessment following GRADE
