# Supplementary figures and images for: Effectiveness of pharmacological procedural sedation in children with cerebral palsy undergoing botulinum toxin injection: a systematic review and meta-analysis
Source: Front Pediatr. 2025 Sep 3;13:1610064. doi: 10.3389/fped.2025.1610064 (PMC12440712; doi:10.3389/fped.2025.1610064)

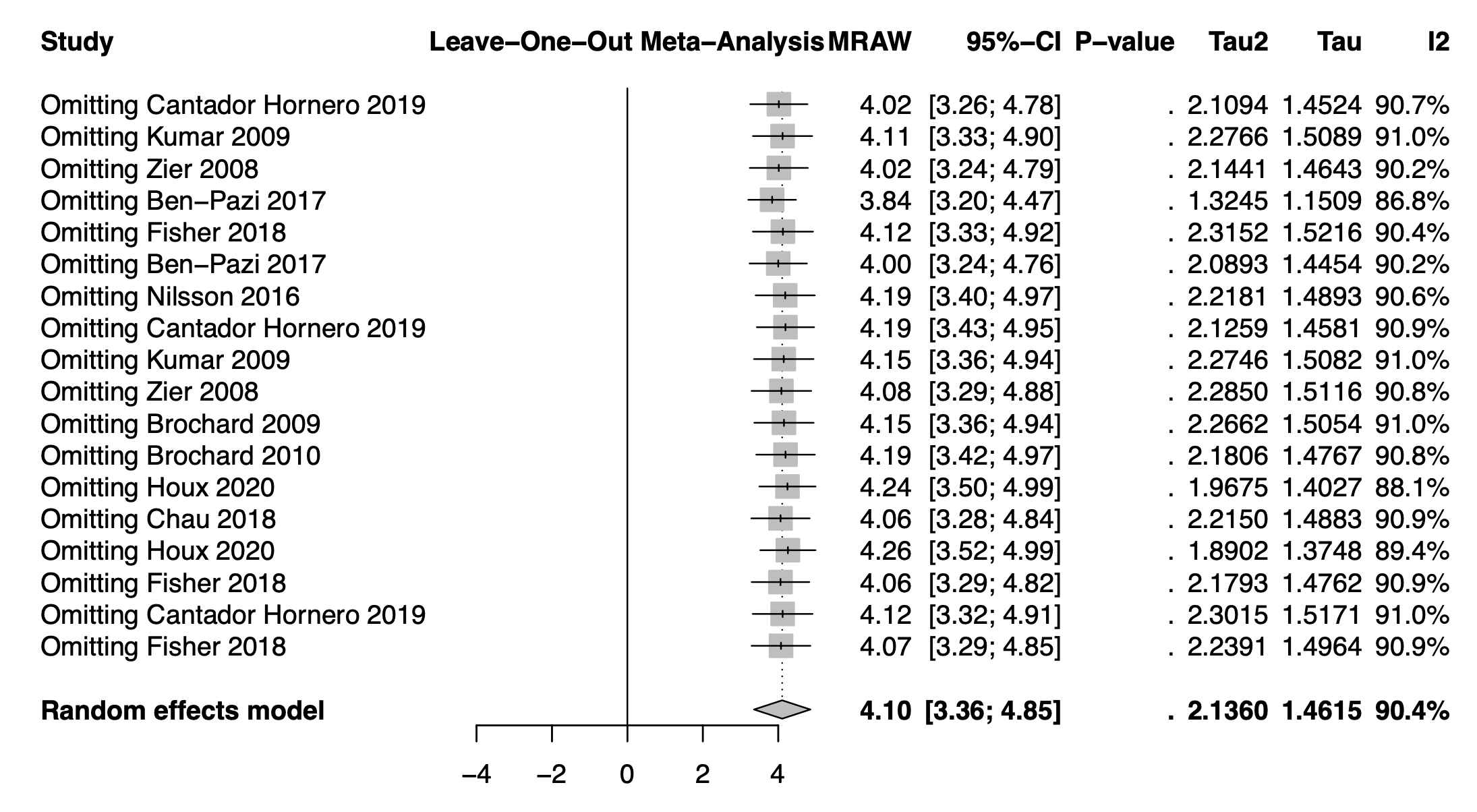

Supplement: Supplementary file 3 [file Image1.png]
